# Curved geometric-phase optical element fabrication using top-down alignment

**DOI:** 10.1515/nanoph-2024-0773

**Published:** 2025-02-14

**Authors:** Gayeon Park, Minseok Kim, Kanghee Won, Seok Ho Song

**Affiliations:** Department of Physics, Hanyang University, Seoul 04763, Republic of Korea; Department of Future Information Display, Kyung Hee University, 26 Kyungheedae-ro, Dongdaemun-gu, Seoul, Republic of Korea; Tigernics, Inc., Seoul, 04763, Republic of Korea

**Keywords:** geometric phase optical element, curved, top-down alignment

## Abstract

Advanced optical technologies, such as next-generation displays and holographic systems, demand high efficiency, lightweight designs, compact dimensions, and compatibility with curved and thin substrates. However, current optical devices for virtual and augmented reality displays, such as surface relief and holographic gratings, face challenges like light scattering, low optical efficiency, and limited scalability. Here, we present a geometric phase optical element (GPOE) fabricated using geometric-phase modulation. A pixelated nano grating system was employed, and reactive mesogens were aligned and transferred via a top-down imprinting process. The resulting GPOE exhibits a thin profile, compatibility with curved surfaces, and scalability for mass production. Integrating additional optical components, we realized a multi-focal GPOE and validated its optical performance. This innovation highlights the potential of GPOEs as compact, next-generation optical components for advanced curved-surface systems.

## 1 Introduction

### 1.1 Background and motivation

With the development of science and technology, we are entering an era characterized by the miniaturization and high performance of electronic components. The growing need for ultrafast information processing and increased data storage capacity has driven the advancement of high-efficiency, compact, and multifunctional devices.

Specifically, next-generation displays require optical components that are highly efficient, compact, and lightweight, while being adaptable to both flat and curved form factors. To meet these demands, significant research efforts have been directed toward developing reflective planar optical devices capable of operating across a wide spectrum, as opposed to conventional transmissive planar systems [1], [2]. This includes studies on holographic surface relief gratings [3], [4], advancements in mixed reality headsets employing holographic optical elements [5], and patent filings targeting the integration of such technologies into three-dimensional head-mounted displays [6]. However, traditional optical components like surface relief and holographic gratings face critical limitations, such as complex fabrication processes and difficulties in accurately shaping intricate wavefronts, which hinder their scalability and commercialization [7], [8]. Although meta-atoms and metasurfaces have been explored to engineer advanced optical functionalities, their transition to commercial applications remains unrealized [9], [10].

Conversely, research on liquid crystal-based geometric phase optical elements (GPOEs) is advancing rapidly, establishing them as promising candidates for next-generation optical devices [11], [12]. In contrast to conventional diffractive optical elements (DOEs), which modulate the optical path through variations in thickness and refractive index, GPOEs rely on anisotropic media whose optical properties are governed by orientation, enabling phase modulation based on the optical axis direction [13], [14]. This capability provides notable advantages for the design of thin, planar, and curved optical devices. Furthermore, GPOEs can suppress scattering from discontinuous surface features, thereby minimizing optical noise and energy loss, which is pivotal for developing compact, lightweight, and high-efficiency devices [15], [16]. While significant efforts have been devoted to planar device research [17], [18], [19], a substantial gap remains in studies focused on curved devices, which are essential for applications such as flexible displays.

### 1.2 Geometric phase and GPOE principles

Conventional optical devices manipulate light through mechanisms like reflection, refraction, and diffraction, achieving phase shifts by altering the material’s thickness and refractive index. By contrast, dynamic phase devices, including beam steering technologies such as beam deflectors (BDs) and liquid crystal lenses, regulate spatial optical path variations through dynamic phase modulation [20], [21], [22]. High resolution BDs capable of large-area operation and wide steering angles are critical for a broad range of applications [23], [24]. Nevertheless, despite their advantages, realizing multifunctional planar optical devices with enhanced performance continues to present substantial challenges [25].

The geometric phase utilizes phase modulation driven by the orientation of the optical axis within an anisotropic medium, controlled by spatial polarization variation. This approach enables the development of thin, planar, and highly efficient devices with continuous phase transitions and minimal optical noise throughout the device.

In anisotropic media, phase retardation (Γ) can be induced by adjusting the refractive index difference (*n*_*e*_ − *n*_*o*_), thickness (*d*), laser wavelength (*λ*), and optical axis angle (*α*). It is expressed as Γ = $\frac{2\pi }{\lambda }\left(n_{e}-n_{o}\right)d\pm 2\alpha$ . The implementation of geometric phase relies on satisfying the half-wave plate condition. When this condition is met, the phase retardation becomes dependent on the optical axis orientation of the anisotropic medium.

Using the Jones matrix (*J*), a matrix representation to describe the polarization state of light, the behavior of light incident on the GPOE can be analyzed [26]. The Jones matrix representing the geometric phase is expressed as *J* = *R*(−*α*) *W R*(*α*), where the phase retarder of the anisotropic medium with rotation angle *α* is expressed as the product of the rotation matrix *R*(*α*) = [cos*α* −sin*α*; sin*α* cos*α*] and the phase retardation matrix *W*(Γ) = [exp(−*i*Γ/2) 0; 0 exp(*i*Γ/2)]. Therefore, the Jones matrix of the GPOE is *J* = cos(Γ/2) I – sin(Γ/2) [cos2*α* sin2*α*; sin2*α* −cos2*α*]. Using this, the Jones vector $E_{\text{in}}=\frac{1}{\sqrt{2}}\left[\begin{array}{c} 1\\ \pm i \end{array}\right]=\chi^{\left(\pm \right)}$ represents the incident right and left circularly polarized light. The output circularly polarized light (*E*_out_) is given by $E_{\text{out}}=J\cdot E_{\text{in}}=\cos\frac{\Gamma }{2}E_{\text{in}}-i\sin\frac{\Gamma }{2}{\text{e}}^{\pm \text{i}2\alpha }\chi^{\left(\mp \right)}$ . This expression includes an additional geometric phase term for the opposite circular polarization, allowing the calculation of the polarization conversion efficiency (*η*) due to the GPOE, $\eta =\sin^{2}\frac{\Gamma }{2}$ . Thus, the polarization conversion efficiency is influenced by the phase retardation of the anisotropic medium, indicating that the thickness of the anisotropic medium within the GPOE plays a critical role in determining the conversion efficiency.

A geometric-phase lens (GPL) is realized based on the principles of geometric phase [27]. When the GPL is fabricated with a thickness meeting the half-wave plate condition, collimated linear polarized light results in half of the light converging and the other half diverging at the focal point. However, if this thickness criterion is not satisfied, part of the light bypasses geometric phase formation and propagates without phase modulation. Consequently, GPOEs can be engineered through this mechanism.

## 2 Design of GPOE

### 2.1 Field tracing-based GPOE design

Using VirtualLab Fusion, the topology profile of the GPOE can be precisely designed specifically, for a focal length of 500 mm at a wavelength of 547 nm, a phase profile from −*π* to *π* can be designed according to the position of the horizontal axis (−2.5 mm to 2.5 mm). The designed GPOE, a GP lens utilizing geometric-phase from the phase retardation of anisotropic media, has the parameters listed in Table 1.

**Table 1:** GPOE parameters used by VirtualLab.

Parameter	Design value	Note
RM refractive index	1.543/1.68	Long axis/short axis
Thickness of RM	1.996 μm/998 nm	Half/quarter wave plate
Focal length of lens	500 mm	–
Maximum phase delay difference	3.14 rad	–
Discrete phase level	512	–
Designed GPOE resolution	10 μm	Nano-grating array size
Wavelength	547 nm	–

### 2.2 Multifocal GPOE lens design

In this research, we integrated a GPOE, designed based on circular polarization conversion efficiency (*η*), with a single-focus lens to observe its multifocal characteristics. The GPOE generated focal points at *f*1, *f*2, and *f*3. When linear polarized light interacted with the GPOE, the portion of light that was not converted formed a focus at the focal length of the single-focus lens (*f*2), whereas the converted light produced focal points at *f*1 and *f*3, separated by the focal length associated with the GPOE. The multifocal behavior of the GPOE lens was found to vary with the circular polarization conversion efficiency, which we demonstrated by designing the GPOE with thicknesses of *λ*/2 (1.996 μm) and *λ/*4 (998 nm). We utilized the Badal lens system [28] to model the optical setup and evaluate lens performance, with the optical components of the Badal lens system summarized in Table 2.

**Table 2:** Badal lens system design value in VirtualLab.

Optical element	Design value
LED	547 nm single wavelength
Target	25 μm pinhole
Badal lens	*f* = 155 mm
Pupil	4 mm
GPL	*f* = 500 mm
Single-focus lens	*f* = 50 mm, plano – convex lens
Detector	1,360 × 1,024 pixels (pixel size = 6.45 μm)

The performance of the designed GPOE lens was evaluated using the parameter run feature in VirtualLab. In particular, the distance between the Badal lens and the target was varied in 1 mm increments from 0 mm to 300 mm, enabling the assessment of lens performance as the focus transitioned across different focal regions. Lens performance was determined by measuring the through focus-point spread function (TF-PSF), which depicts the light intensity along the direction of propagation, and the through focus-modulation transfer function (TF-MTF), which evaluates lens resolution based on focus region shifts at a given spatial frequency. These evaluations were performed using PSF and MTF detectors.

## 3 Fabrication of GPOE

### 3.1 GPOE fabrication process

The conventional fabrication of GPOEs utilizes a bottom-up alignment approach, requiring an internal alignment layer within the device structure [1], [12]. In this research, we introduce a top-down alignment method that eliminates the need for such an alignment layer, enabling the development of ultra-thin GPOEs.

To achieve this, we utilize reactive mesogens (RMs, RMS03-13, Δ*n* ∼ 0.137, Merck), a type of liquid crystal with rod-like molecular structures and photoresponsive functional groups, exhibiting anisotropic properties due to their oriented optical axis [29]. The top-down alignment method allows precise control of the optical axis in the anisotropic medium by employing an alignment layer directly applied to the device surface, effectively addressing the thickness and efficiency constraints of conventional GPOEs. Importantly, by aligning the RM through a top-down approach using an alignment layer on a poly dimethyl siloxane (PDMS) stamp, this method enables the fabrication of GPOEs on curved substrates, paving the way for optical devices with diverse form factors.

The fabrication of GPOEs, which utilize geometric phases for modulating light phase and transforming polarization, necessitates precise alignment techniques. RM alignment can be accomplished through the creation of geometric grooves using the rubbing method [30] or by employing photoalignment techniques that leverage liquid crystal alignment derivatives [31], [32].

**Figure 1:**
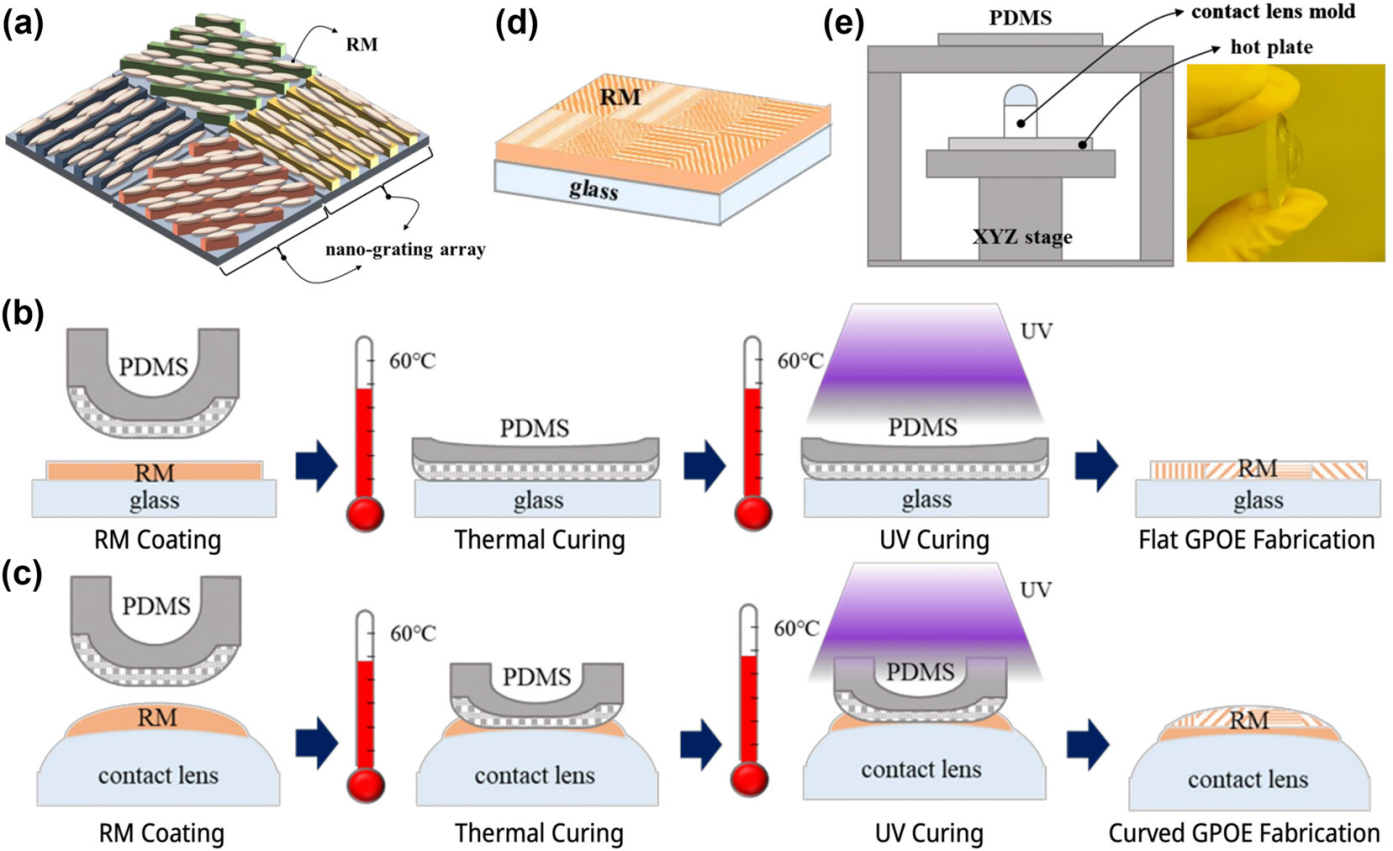
GPOE fabrication process. (a) RM with optical axis oriented by nano grating array. (b) Flat GPOE fabrication process. (c) Curved GPOE fabrication process. (d) Flat GPOE manufactured using top-down alignment. (e) Curved GPOE combined with contact lens.

In this research, we fabricated a nano-grating array with arbitrarily oriented geometric grooves on a Si wafer using a pixelated nano-grating system (Supplementary Figure S1). The RM, patterned through an imprinting process involving resin to create the stamp and replica, is depicted in Figure 1(a). The optical axis of the RM aligns with the nano-grating array, rendering the fabrication process simple and cost-effective, making it suitable for large-scale production [33].

As shown in Figure 1(b)–(e), a flat GPOE was fabricated on a glass substrate, while a curved GPOE was constructed on a contact lens. Both types were achieved by aligning the RM using a PDMS stamp. The flat glass substrate simplifies RM alignment, whereas the curvature of contact lenses enables RM alignment to conform seamlessly to curved surfaces. Consequently, the method proposed in this work enables the fabrication of both flat and curved GPOE structures.

### 3.2 Stamp fabrication process

To fabricate the GPOEs presented in this paper, the production of a stamp is crucial. This process can be broadly divided into three main stages: creating a master substrate (Supplementary Figure S2) with a nano-grating array on a Si wafer, producing a resin stamp (Supplementary Figure S3), and fabricating a PDMS stamp (Supplementary Figure S4).

The fabrication of the master substrate comprises multiple steps, including Si wafer patterning, spin coating, baking, and exposure. In particular, a pixelated nano-grating system is utilized to create the nano-grating array on the Si wafer. To minimize surface reflections, a bottom anti-reflective coating is applied, followed by the spin coating and baking of a photoresist (PR) layer. During the exposure step, a grating wafer with sub-micron lens phase elements is produced.

For the resin stamp, the master substrate undergoes ozone treatment to make the surface hydrophilic. During the imprinting process, resin is applied to replicate the nano grating pattern. Detailed procedures involve configuring the height, speed, and start/stop positions of the roller in a wafer laminating system to achieve uniform resin coating on the master substrate, followed by curing under a 365 nm ultra violet (UV) lamp. The cured resin is then transferred to a PET film, and upon separation from the master substrate, a resin stamp containing the nano-grating array is obtained.

Finally, a resin stamp is employed to create a PDMS stamp embedded with the nano-grating array. The process begins with vacuum-treating the PDMS in a desiccator for over 30 min to remove air bubbles, preserving the fidelity of the grating pattern. Liquid PDMS is then poured into a petri dish, ensuring the resin stamp’s grating pattern faces upward. A second vacuum treatment is performed to eliminate any remaining air bubbles, followed by heat curing of the PDMS. Once solidified, the PDMS is carefully detached from the resin stamp, resulting in a PDMS stamp with the nano grating array.

### 3.3 Top-down alignment for GPOE fabrication

#### 3.3.1 Novelty and advantages over existing methods

The fabrication methods for the GPOEs presented in this paper can be categorized into two key processes: the creation of nano-gratings using a pixelated nano-grating (PNG) system and the alignment of RMs via a top-down alignment method, which involves transferring the nano-grating patterned alignment layer. These techniques enable the production of embedded GPOEs.

Prior to the advent of GPOEs, optical devices such as lenticular lenses commonly relied on a combination of bottom-up and top-down alignment methods for liquid crystal orientation [34]. Similarly, the top-down alignment method has been applied to align RMs in micro-lens fabrication [35]. These alignment strategies have been extensively studied, with continued efforts to implement either top-down or bottom-up approaches for liquid crystal alignment even after the development of GPOEs.

In the fabrication of GPOEs, methods such as photoresist engraving have been employed to ensure the alignment of RMs with the grating grooves [14]. Additional techniques, including photoalignment [36] and micro-rubbing [37], have been utilized to create liquid crystal arrays. However, these approaches often necessitate extra processing steps and result in thicker devices when using the bottom-up alignment method. The introduction of the pixelated nano-grating system, as utilized in this study, overcomes these limitations. The nano-pixelated grating system, developed in previous research, facilitates the cost-effective, large-scale production of GPOEs.

The PNG system consists of an interferometer-based optical setup at the top and a movable stage at the bottom. By adjusting the angle and horizontal azimuth of the two laser beams in the upper setup, the system enables precise control over the grating period and orientation for each grating pixel, allowing for rapid pattern generation. Previous studies have showcased the high-speed fabrication of large-area GPOEs using the PNG system in combination with micro dot rubbing [38], and the integration of these elements with lenses has facilitated the development of multi-focal GPOEs [39]. Current research efforts aim to advance compact optical devices by fabricating multi-focal lenses within a single layer, eliminating the need for multiple layers [40].

The key difference between this novel approach and conventional flat GPOE methods lies in employing the PNG system to achieve top-down alignment for RM orientation. By integrating the traditional PNG system with the top-down alignment technique, this method eliminates the need for internal alignment layers, resulting in thinner GPOEs. Furthermore, this approach facilitates the fabrication of GPOEs in thin-film form, making them suitable for a wide range of curved optical devices. Therefore, this approach enables the formation of a GP lens on any curved surface. Additionally, the proposed top-down method has an advantage for mass production since any additional alignment layer normally used in a bottom-up approach is not required. Quantitatively, in the bottom-up alignment method, a residual resin layer is formed during the patterning process on the substrate. Due to the nature of the hand-rolling process, this residual layer typically results in an additional thickness of approximately 3–50 μm. In contrast, the top-down alignment method eliminates the formation of any residual resin layer, thereby completely avoiding such thickness increases. Consequently, the top-down method achieves a notable reduction in overall thickness.

#### 3.3.2 Curved GPOE fabrication method

To fabricate a curved GPOE using a contact lens substrate for RM alignment, the contact lens was utilized in its dry, pre-hydration state while retained within its original mold. A custom alignment jig (Supplementary Figure S5) was designed to securely position the PDMS stamp and substrate, ensuring precise centering of the contact lens along the *X*–*Y* axis and enabling *Z* axis adjustments to bring the contact lens into contact with the PDMS stamp. For thermal curing, the stage was equipped with a hot plate, as depicted in Figure 1(e).

**Figure 2:**
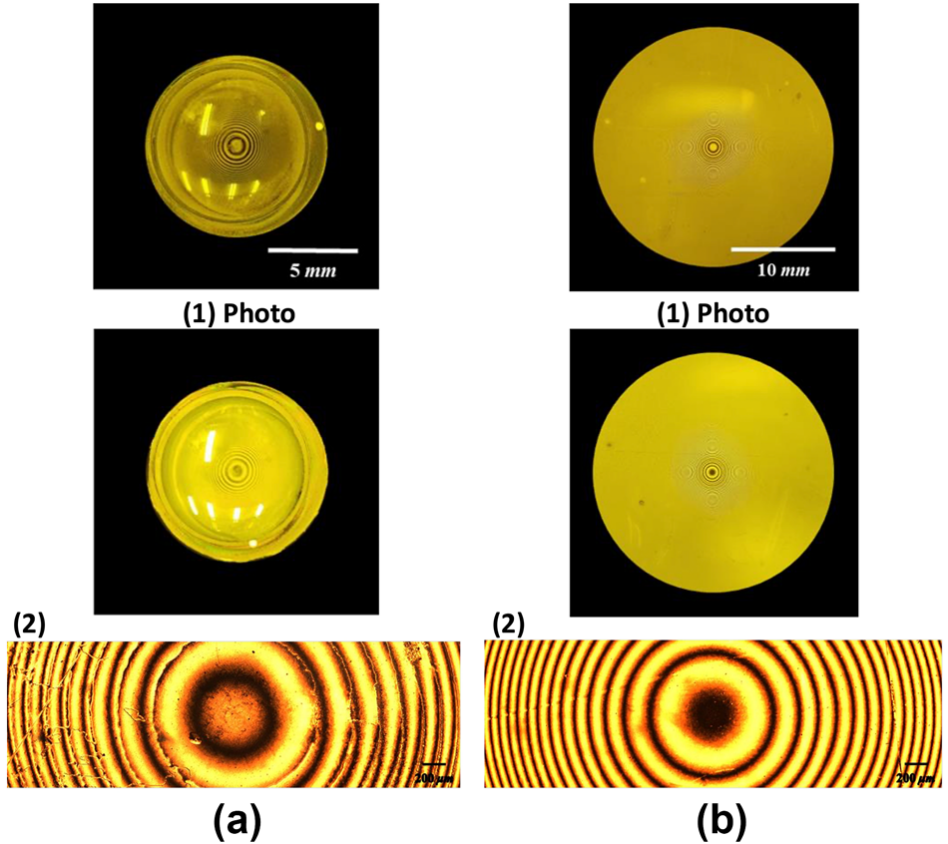
Produced GPOE. (1) Photo and (2) 50× maginification microscope image. (a) Curved GPOE fabricated between two crossed polarizers. (b) Flat GPOE fabricated between two crossed polarizers.

During the fabrication process, RM was applied to the contact lens, brought into contact with the PDMS stamp, and thermally cured using the integrated hot plate. This was followed by UV exposure for additional curing. After separating the PDMS stamp from the contact lens, a curved GPOE was obtained, with the RM aligned to the nano-grating pattern on the PDMS stamp. The RM thickness was controlled by adjusting the spin coating speed and duration. Additionally, precise control of the spacing and applied force between the PDMS stamp and the contact lens was crucial to prevent alignment errors. When the applied force is relatively weak, the PDMS stamp does not make full contact with the soft contact lens, resulting in partial alignment of the RM layer. Additionally, variations in the force exerted by the PDMS stamp at each position lead to non-uniform RM thickness. Conversely, when the applied force is excessively strong, the PDMS stamp may sustain damage, potentially tearing during the experiment. In this case, the PDMS stamp fully contacts the soft contact lens, resulting in overall uniform RM thickness. Therefore, it is essential to apply an appropriate amount of force to ensure that the PDMS stamp makes complete contact with the soft contact lens without sustaining damage, thereby achieving uniform RM thickness. The final curved GPOE is presented in Figure 2(a).

This method can also be adapted for the fabrication of a flat GPOE by aligning RM on a glass substrate. The performance of the flat GPOE is strongly affected by the pre-treatment process, which involves cleaning the glass surface to remove contaminants using acetone, alcohol, deionized water, and an ultrasonic cleaner, followed by drying. To enhance surface adhesion, an adhesion promoter is applied via spin coating and thermally cured.

Subsequently, RM is spin-coated onto the pre-treated glass substrate and thermally cured. The PDMS stamp is then aligned and placed on the substrate, with the pattern transferred through a combination of thermal curing and UV exposure. After the PDMS stamp is removed, a flat GPOE is formed with RM aligned to the nano-grating pattern on the PDMS stamp, as shown in Figure 2(b).

## 4 Experimental characterization

### 4.1 Measurement of polarization conversion efficiency

**Figure 3:**
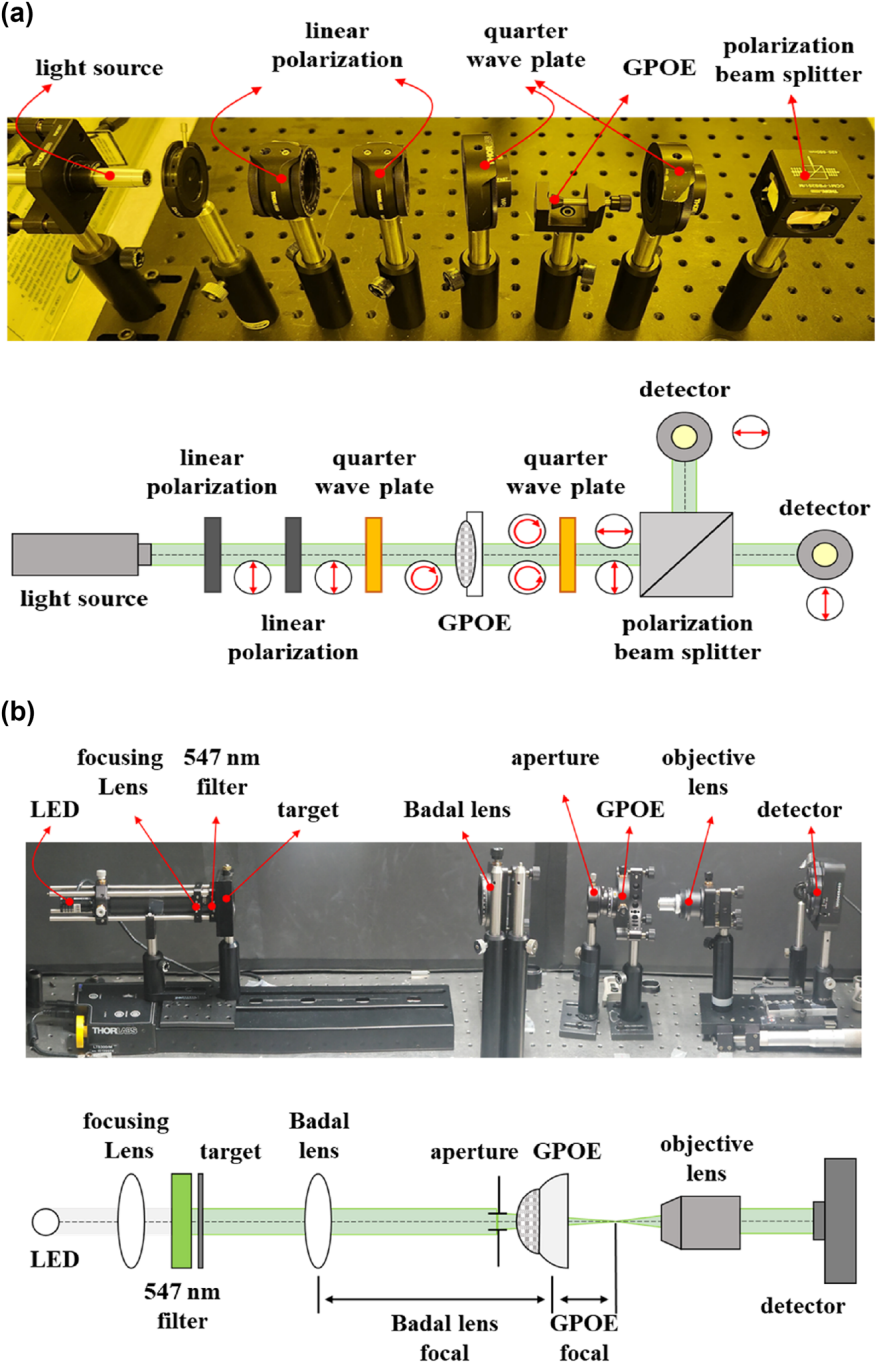
GPOE characteristic measurement system. (a) Polarization convention efficiency measurement. (b) Lens performance measurement of multifocal GPOE.

To evaluate the polarization conversion efficiency (*η*) of the GPOE, circularly polarized light was introduced, and the resulting right-handed and left-handed circularly polarized components were separated using a polarization beam splitter. The measurement setup is depicted in Figure 3(a).

In the optical setup, a 532 nm laser beam first passes through two linear polarizers and a quarter-wave plate (QWP) to produce right-handed circularly polarized light. As the light propagates through the GPOE, a portion is converted into left-handed circularly polarized light, while the remaining portion retains its right-handed polarization. After passing through a second QWP, the converted beams are transformed into horizontal and vertical linear polarizations, respectively. These polarizations are subsequently separated by a polarization beam splitter and measured with a power meter. The thickness of the RM is then determined using the formula for circular polarization conversion efficiency.

### 4.2 Measurement of multifocal GPOE lens performance

To integrate the GPOE fabricated in this study with a single-focus lens, UV resin and a UV lamp were used to produce a multifocal GPOE, as shown in Figure 1(e). To evaluate the lens performance of the multifocal GPOE, a measurement system incorporating a Badal lens was employed. This system maintains a constant target image size across varying focal lengths, offering a distinct advantage for performance assessment, as illustrated in Figure 3(b).

The GPOE lens performance measurement system consists of a white LED, focusing lens, 547 nm filter, target, plano-convex lens (*f* = 50 mm), GPOE, objective lens, and detector. The objective lens provides 10× magnification for TF-PSF and TF-MTF measurements, and 4× magnification for TF-image measurements. The light source and target are mounted on a linear stage that can scan distances from 0 mm to 300 mm. By adjusting the distance between the target and the Badal lens, TF-PSF, TF-MTF, and TF-image measurements can be conducted as the focal length varies.

Specifically, the TF-PSF and TF-MTF measurements utilize a 25 μm pinhole target. By scanning the distance between the target and the Badal lens from 0 mm to 300 mm in 1 mm increments, the TF-PSF can be recorded based on the intensity detected by the detector. This data is then Fourier transformed to obtain the complex optical transfer function (OTF), which characterizes the image response of the optical system. The real part of the OTF represents the modulation transfer function (MTF), and the imaginary part corresponds to the phase transfer function (PTF), allowing the MTF to be determined by taking the magnitude of the OTF [41], [42].

For TF-image measurements, a 1951 United States air force (USAF) resolution target is employed. The scanning procedure remains the same as previously described, with target images captured at different distances to evaluate performance. By analyzing these images, the imaging capabilities of the system can be assessed.

### 4.3 Analysis of GPOE lens characteristic results

In this research, we scanned the focal length of the fabricated multifocal GPOE lens to measure TF-PSF, TF-MTF, and TF-image, and compared the results with the design specifications. Through the evaluation of polarization conversion efficiency, we achieved GPOE devices with a conversion efficiency of 92 % for a *λ*/2 thickness and 47 % for a *λ*/4 thickness. Considering the single-focus lens with a focal length of 150 mm, we analyzed the performance of the multifocal GPOE lens and verified that it exhibited bifocal and trifocal characteristics, using 150 mm as the reference.

#### 4.3.1 Evaluation of GPOE TF-PSF and TF-MTF result

**Figure 4:**
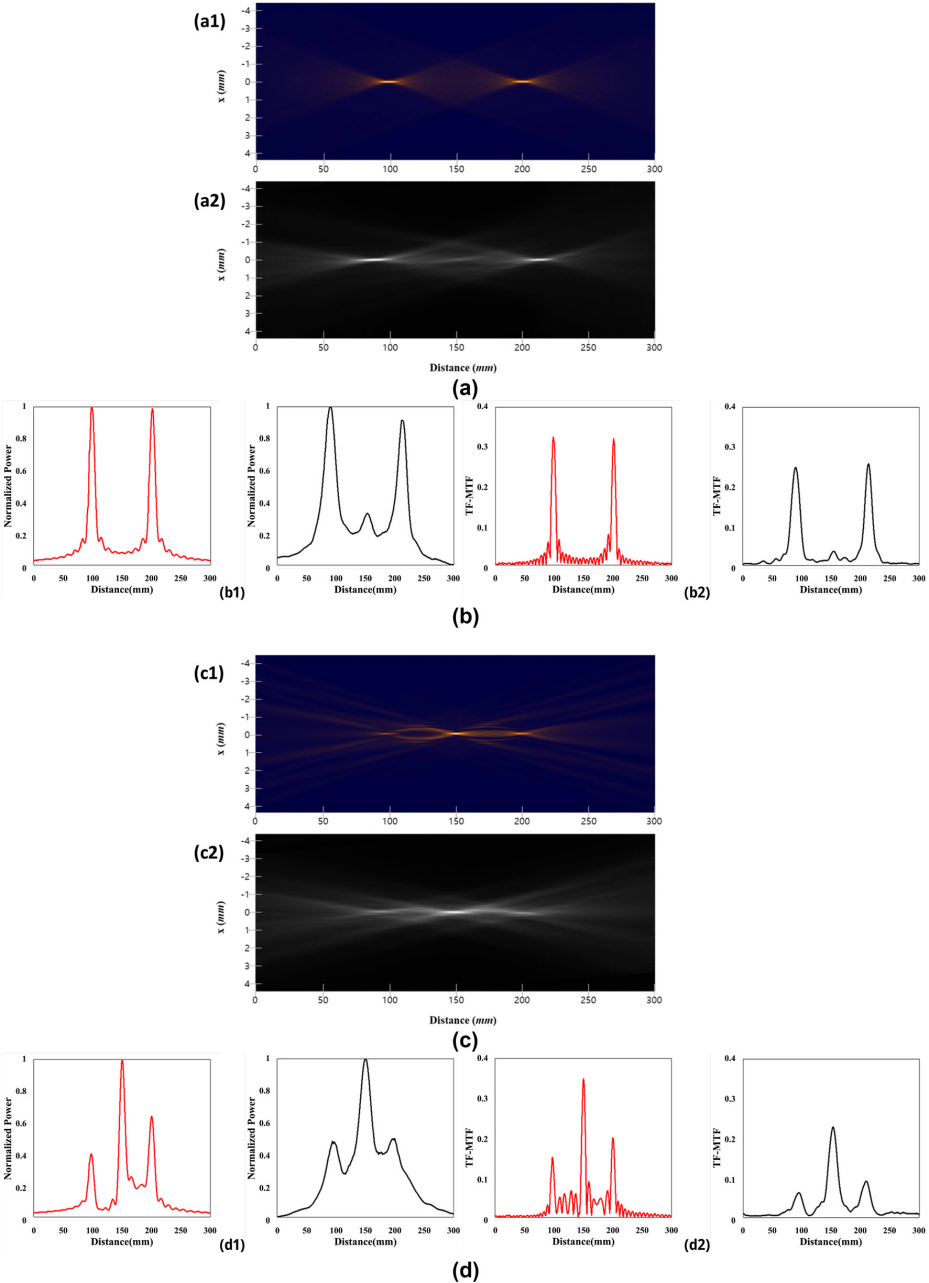
Analysis of the results of GPOE TF-PSF, TF-MTF. (a, b) Double focus GPOE lens with a *λ*/2 thickness. (c, d) Triple focus GPOE lens with a *λ*/4 thickness. (a) TF-PSF design values (1) and measurements (2) by distance. (b) Normalized power distribution design values (shown on the left), measurements (shown on the right) along the propagation axis at *x* = 0 (1) and TF-MTF design values (shown on the left) and measurements (shown on the right) at space frequency 50 line pairs/mm (2). (c) TF-PSF design values (1) and measurements (2) by distance. (d) Normalized power distribution design values (shown on the left), measurements (shown on the right) along the propagation axis at *x* = 0 (1) and TF-MTF design values (shown on the left) and measurements (shown on the right) at space frequency 50 line pairs/mm (2).

A GPOE with a *λ*/2 thickness achieves a polarization conversion efficiency of 92 %. Light transmitted through this GPOE is converted into right-handed and left-handed circularly polarized components with 92 % efficiency, forming a bifocal lens. Experimental measurements verified the creation of two focal points, consistent with the design values depicted in the TF-PSF data (Figure 4(a)).

For TF-MTF (Figure 4(b)), the normalized power distribution along the propagation axis at *X* = 0 and the TF-MTF design values at a spatial frequency of 50 line pairs/mm both exhibit two distinct peaks, demonstrating the bifocal characteristics of the GPOE. The experimental measurements likewise revealed two peaks, further validating the bifocal of the device.

A GPOE with a thickness of *λ*/4 shows a polarization conversion efficiency of 47 %. The light passing through this GPOE is converted into right-handed and left-handed circularly polarized light with 47 % efficiency, resulting in a trifocal lens. Similar to the *λ*/2 GPOE, the TF-PSF measurements (Figure 4(c)) confirmed the presence of three focal points, as indicated by the design values. The TF-MTF (Figure 4(d)) design and measurement values both showed three peaks, confirming the trifocal nature of the GPOE.

These results clearly demonstrate that the *λ*/2 and *λ*/4 GPOEs operate as bifocal and trifocal lenses, respectively, as evidenced by their polarization conversion efficiencies and the focal points observed in the TF-PSF and TF-MTF measurements.

#### 4.3.2 Evaluation of TF-image results

**Figure 5:**
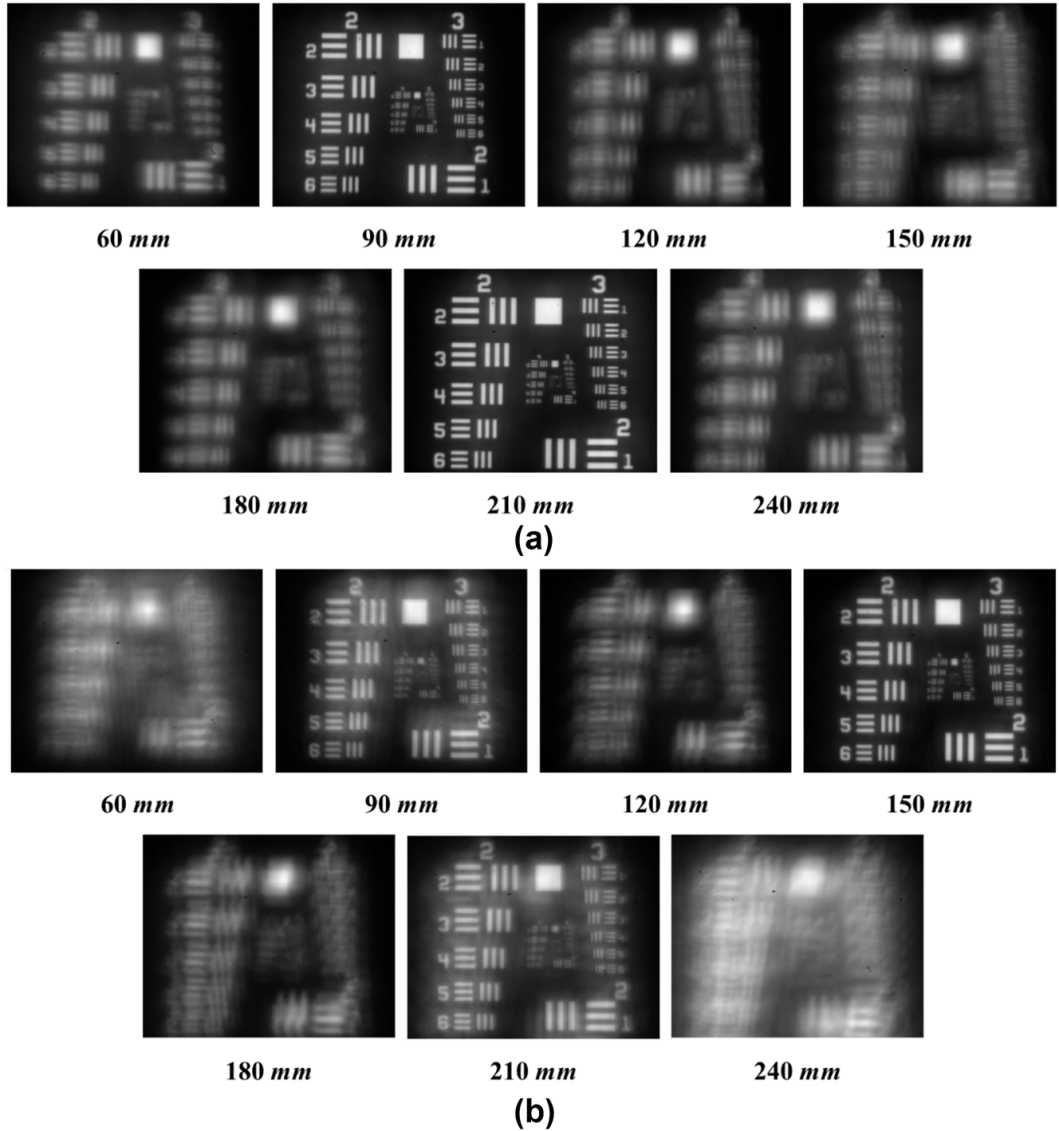
GPOE TF-image results. (a) Double focus GPOE lens with a *λ*/2 thickness. (b) Triple focus GPOE lens with a *λ*/4 thickness.

In this research, the TF-image was measured using a 1951 USAF Resolution Target, as depicted in Figure 5, to evaluate the performance of the GPOE lens. Since the image at 150 mm corresponds to the focal length of a single-focus lens, assessing the focal point at different positions enables the evaluation of the multifocal lens performance.

In Figure 5(a), for the GPOE with a *λ*/2 thickness, the TF-image measurements at various target distances revealed focal points at 90 mm and 210 mm. This result verifies that the GPOE operates as a bifocal element, producing two distinct focal points.

Similarly, in Figure 5(b), for the GPOE with a *λ*/4 thickness, the TF-image measurements indicated focal points at 90 mm, 150 mm, and 210 mm. This demonstrates that the GPOE functions as a trifocal element, forming three distinct focal points.

#### 4.3.3 Discussion of experimental results

In this experiment, TF-PSF, TF-MTF, and TF-image measurements were conducted using the fabricated GPOE. By comparing and analyzing the design values with the experimental results from devices of different thicknesses, we confirmed the multifocal characteristics of the GPOE. However, a discrepancy was observed between the designed efficiency and the actual efficiency of the fabricated GPOE. This difference is likely attributed to scattering caused by misaligned regions of the RM during the fabrication process, resulting in optical axis misalignment between the GPOE and the single-focus lens.

To resolve this issue, achieving more precise alignment of the RM is essential. Improving the alignment accuracy will help minimize scattering, thereby enhancing the overall performance and efficiency of the GPOE, bringing it closer to the intended design specifications.

## 5 Conclusions

In this paper, we demonstrated the fabrication of GPOEs with thin profiles on curved substrates using a top-down alignment method. During the fabrication process, a master substrate was fabricated as a nano-grating array using a pixelated nano-grating system based on light interference phenomena. The pattern from the master substrate was transferred through an imprinting process to create resin and PDMS stamps. These stamps were then employed to fabricate GPOEs on contact lens substrates via top-down alignment. Moreover, this technique can also be applied to produce flat GPOEs.

Microscopic images of the fabricated GPOEs verified the successful alignment of the RM using the nano-grating array structure through the top-down alignment method. When combined with a single-focus lens, the GPOEs displayed multifocal characteristics. Additionally, through a lens performance measurement system, we assessed TF-PSF, TF-MTF, and TF-image. The findings confirmed that the GPOEs produced using the top-down alignment method were thin and possessed the intended optical properties.

The compact GPOE proposed in this paper holds significant potential for various applications. For example, next-generation optical systems for advanced displays require high-efficiency optical components. Although holography offers a potential solution to the vergence-accommodation conflict (VAC) in current VR/AR devices, its implementation remains challenging due to existing technological limitations. As a practical alternative, GPOEs could be employed to create multiple focal planes on a single surface, effectively addressing the VAC issue. Moreover, current holographic video displays already utilize GPLs [43], which offer advantages over traditional optical components in terms of reduced thickness and weight [44]. By incorporating curved GPOE films, further reductions in thickness could be achieved, accelerating the commercialization of mobile holographic displays. Additionally, in applications such as light detection and ranging and other sensors where minimizing bulk is essential, GPOEs can effectively address volume constraints [45], [46]. Furthermore, with the growing need for vision correction due to an aging population, GPOEs could be integrated into intraocular lenses for presbyopia correction and cataract surgery [47]. Conclusively, this innovation is expected to establish itself as a next-generation optical solution, offering broad applicability across diverse fields.

## Supplementary Material

Supplementary Material Details
