# Supplementary material for: Curved geometric-phase optical element fabrication using top-down alignment
Source: Nanophotonics. 2025 Feb 14;14(4):523–34. doi: 10.1515/nanoph-2024-0773 (PMC11834054; doi:10.1515/nanoph-2024-0773)
Supplement: Supplementary file 1 — Supplementary Material Details [file j_nanoph-2024-0773_suppl_001.pdf]

# [Supplementary Information]

# Curved geometric-phase optical element fabrication using top-down alignment

Gayeon Park[1], Minseok Kim[2], Kanghee Won[2,*], and Seok Ho Song[1,3]

[1]Department of Physics, Hanyang University, Seoul 04763, Republic of Korea

[2]Department of Future Information Display, Kyung Hee University, Seoul 02447, Republic of Korea

[3]Tigernics, Inc., Seoul, 04763, Republic of Korea

* Correspondence: khwon@khu.ac.kr

**Contents:**

**S1.** Pixelated nano-grating system

**S2.** Detailed fabrication process for master substrate and stamp

**S3.** Curved geometric-phase optical element (GPOE) manufacturing space

**S4.** Detailed fabrication process for reactive mesogen (RM) alignment

## **Supplementary Note S1:** Pixelated nano-grating system

In this study, a pixelated nano-grating system was utilized for the fabrication of the geometric-phase optical element (GPOE). This system is designed to form nano-scale patterns. Specifically, the nanopatterned surface that forms the nano-grating array using pixelated nano-grating system is composed of square grating pixels (grating period of 500nm) with discrete corrugation angles (ϕ), a constant width (pixel size, p= 5 μm), and a height of up to 120 nm, as shown below Figure S1.

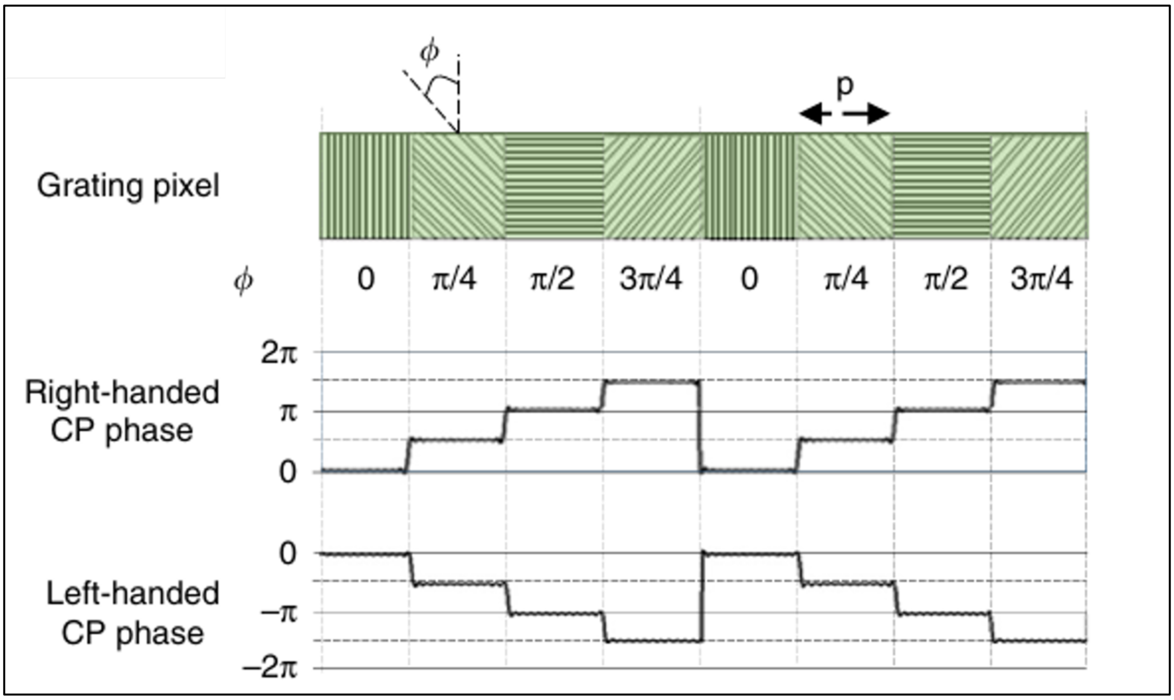


**Figure S1:** Pixelated nano-grating system and nanopatterned surface. The nanopatterned surface is made up of square grating pixels with discrete corrugation angles (*ϕ*) and a constant width (*p*), along with the associated GP modulation (*2ϕ*) for incidences of right-handed and left-handed circularly polarized light.

Additionally, as the corrugation angle (ϕ) varies stepwise along the grating pixels, the left-handed circular polarization (LCP) coated on the alignment layer transitions from an inhomogeneous in-plane distribution, following the ϕ directions, to a discrete configuration with a full

alignment range of $\phi$ = 0°–180°. The angle resolution is defined as $\Delta\phi$ = 180° / 4096 (12-bit), corresponding to 0.044°. The right-handed circular polarization (RCP) component of light, after passing through the aligned LCP layer, undergoes a phase shift of 0–2$\phi$, while the LCP component exhibits the opposite behavior.

## **Supplementary Note S2:** Detailed fabrication process for master substrate and stamp

This section provides a detailed explanation of the fabrication process for the master substrate and stamp, which was not extensively covered in Section 3 (Fabrication of GPOE) in the main manuscript.

*[Master Substrate]*

Using the pixelated nano-grating system, a silicon (Si) wafer was fabricated as the master substrate with a nano-grating array. To reduce surface reflection, bottom anti-reflective coating (BARC, Brewer Science, DUV252-310) and photoresist (AZ Electronic Materials, AZ 5206-E) were spin-coated onto the Si wafer. BARC was applied using a 0.45 μm syringe filter, followed by baking at 195 °C for 1 minute on a hot plate and cooling at room temperature for 1 minute. The photoresist was then applied using a 0.1 μm syringe filter, baked at 110 °C for 1 minute on a hot plate, and cooled at room temperature for 1 minute. After completing the baking process, the Si wafer was exposed using the pixelated nano-grating system by interfering two beams to fabricate a wafer with sub-micron lens-phase gratings. Figure S2 shows an image of the fabricated master substrate.

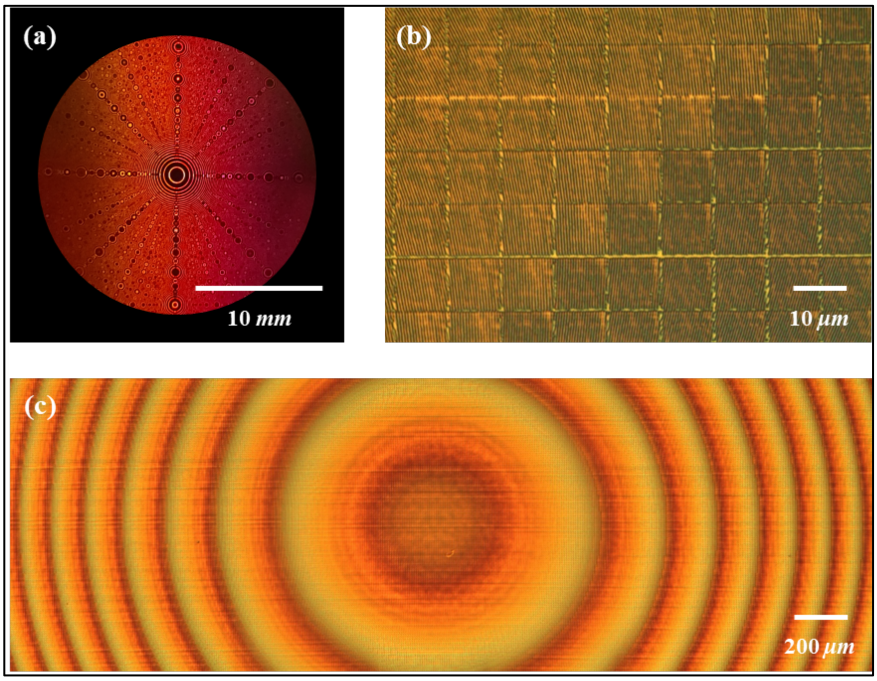


**Figure S2:** Images of the master substrate with a nano-grating array. (a) Photograph of the master substrate, (b) x1000 magnification microscope image, (c) x50 magnification microscope image.

*[Resin Stamp]*

Based on the fabricated master substrate, a resin stamp with a nano-grating array pattern was created using an imprinting process. To produce the resin stamp, the master substrate was treated with ozone using an ozone cleaner (Omniscience Co.) for 10 minutes to render the surface hydrophilic. Subsequently, an ionizer was used for 30 seconds to remove dust from the master substrate.

The imprinting process was then carried out using a wafer laminating system (Hantech Co.). The master substrate was placed at the center of the wafer laminating system stage, and 1 mL of resin (Minuta Tech Co., MIN-311RM) was applied onto the substrate. A PET film (KAPS Co.) larger than the master substrate was cut and placed over the substrate. The roller height, speed, starting position, and ending position of the wafer laminating system were adjusted to

ensure the resin was evenly distributed across the entire surface of the master substrate. After the roller passed over, the resin was cured by exposing it to 365 nm UV light using a UV lamp (2SD Co., Linear type UV-LED curing system). The cured resin was transferred onto the PET film. Finally, the ionizer was used to separate the master substrate and the PET film while removing any dust. Figure S3 shows the image of the resin stamp with a nano-grating array pattern formed on the separated PET film.

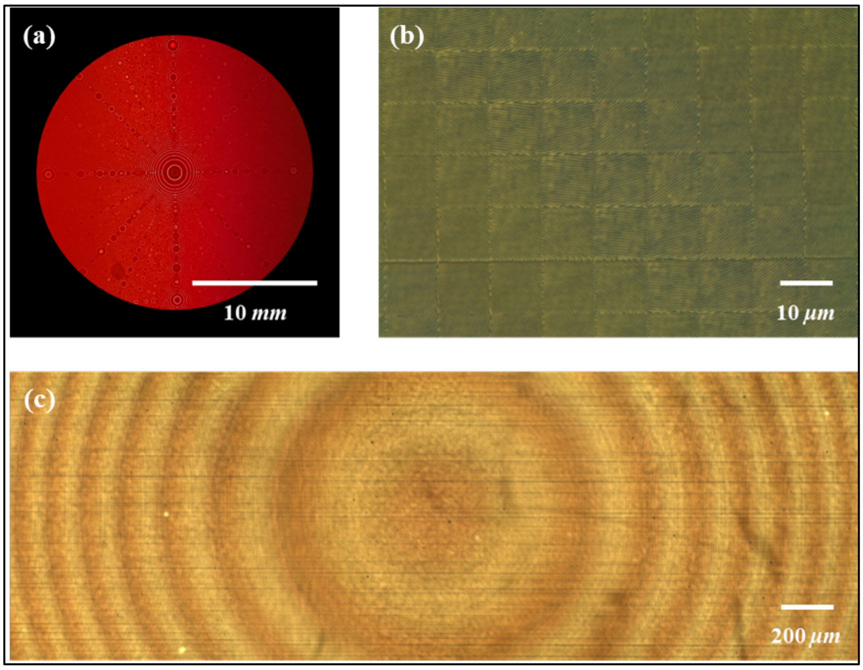


**Figure S3:** Images of the resin stamp with a nano-grating array. (a) Photograph of the resin stamp, (b) x1000 magnification microscope image, (c) x50 magnification microscope image.

*[PDMS Stamp]*

A PDMS stamp with a nano-grating array pattern was fabricated based on the resin stamp. PDMS (DOW CORNING Co.) was prepared by mixing the base and curing agent at a 10:1 ratio. To remove air bubbles, the mixture was placed in a desiccator under vacuum for more than 30 minutes. A petri dish was used to form the grating pattern on the fluid PDMS. The resin stamp was cut to fit the size of the Petri dish and placed with the grating side facing up. The degassed

PDMS was then poured over the resin stamp. The petri dish containing PDMS was placed back in the desiccator under vacuum for at least 10 minutes to remove any remaining bubbles. Once the bubbles were completely removed, the PDMS was cured on a hot plate at a temperature above 60 °C for more than 1 hour. After curing, the solidified PDMS was separated from the resin stamp. Figure S4 shows the image of the nano-grating array formed on the separated PDMS stamp.

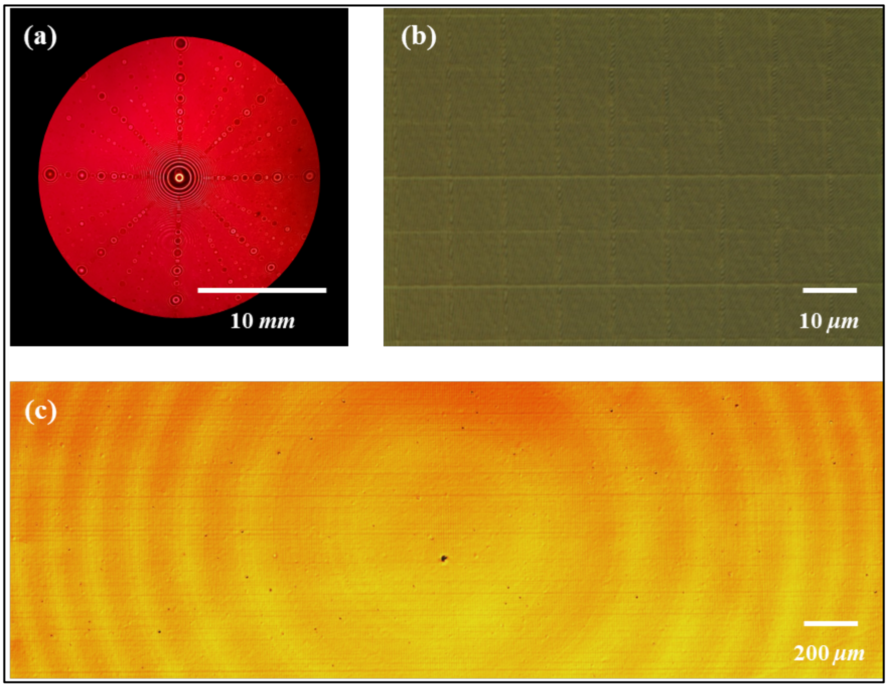


**Figure S4:** Images of the PDMS stamp with a nano-grating array. (a) Photograph of the PDMS stamp, (b) x1000 magnification microscope image, (c) x50 magnification microscope image.

**Supplementary Note S3:** Curved GPOE manufacturing space

Figure 1(e) in Section 3, fabrication of GPOE, of the main text presents a schematic diagram

of the curved GPOE fabrication process. This supplementary section provides additional details to further elaborate on the fabrication process. In the front-view schematic presented in Figure 1(e) of the main text, a barrier is depicted between the XYZ stage and the PDMS stamp. However, this front-view illustration does not fully capture the actual configuration. As shown in the top-view schematic and the accompanying photograph provided below Figure S5, the setup is specifically designed to enable contact between the PDMS stamp and the contact lens after the alignment process is completed.

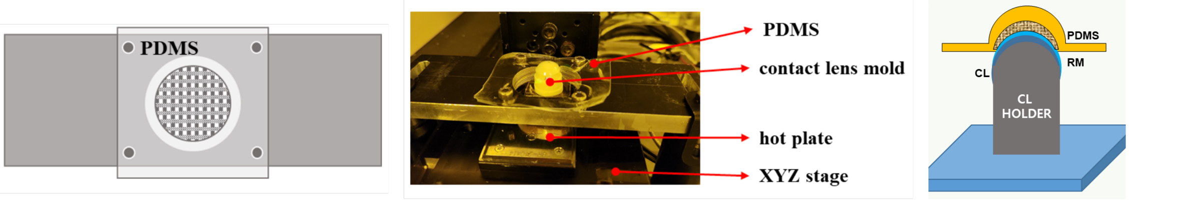


< Top-view > < Actual photograph > < Illustration >

**Figure S5:** Alignment jig for curved GPOE manufacturing. From left to right, the top-view schematic of the alignment jig is shown, followed by an actual photograph of the setup. These visuals demonstrate the configuration and contact mechanism between the PDMS stamp and the contact lens after the alignment is completed.

## **Supplementary Note S4:** Detailed fabrication process for reactive mesogen alignment

The detailed process conditions for aligning the reactive mesogen (RM) and fabricating the curved GPOE using a contact lens are described as follows. The RM (RMS03-13, $\Delta n \sim 0.137$, Merck) was spin-coated onto a contact lens at 1000 rpm for 30 seconds under half wave plate conditions. After coating, the contact lens was placed on a hot plate within an alignment jig, and the z-axis of the jig was adjusted to ensure contact between the PDMS stamp and the

contact lens. Thermal curing of the RM layer was conducted at 60 °C for 1 minute. Following the thermal curing, the RM layer was exposed to UV light (365 nm, 23 mW/$cm^2$) at 60 °C for 5 minutes to complete the curing process. The imprinting process was subsequently performed using a hand roll.
